# Supplementary material for: Bloom syndrome helicase facilitates efficient HIV-1 infection in macrophages
Source: NAR Mol Med. 2026 Oct 22;3(4):ugag047. doi: 10.1093/narmme/ugag047 (PMC13760215; doi:10.1093/narmme/ugag047)
Supplement: Supplementary file 1 [file ugag047_supplemental_files.zip › Revised Supplemental Leal et al NARMME.docx]

**SUPPLEMENTAL MATERIALS**

Supplemental Figures

**Fig S1. Cell viability with DDR inhibitors**

(A) MDMs were treated with DMSO (10 μM) or KU-55933 (ATM inhibitor), olaparib (PARP1 inhibitor), or VE-821 (ATR inhibitor) at indicated concentrations for 72 h. PrestoBlue cell viability reagent was added to media (1:10) and incubated for 1 h before absorbance was read at 570nm and 600nm. Percentages calculated and compared to normalized DMSO control. (B) Total cell DNA was extracted 3 dpi and assed for proviral integration by Alu-PCR. Average calculated integrated provirus per cell for DMSO treated MDMs across each experiment. Data are displayed as the means ± SEM with each dot representing a different MDM donor.

**Fig S2. CRISPR Screen identifies DDR genes influencing HIV-1 infection**

(A) Volcano plot for gene hits generated by plotting -log10(p-values) over log2 fold change (LFC) for GFP-positive cells against GFP-negative cells (supplemental data set 1) (B) Volcano plot for gene hits comparing GFP-positive cells against uninfected cells (supplemental data set 2). (C) Violin plots of LFC for GFP-positive cells against GFP-negative cells and (D) and GFP-positive cells against uninfected cells. Y-axis lines represent ± 5% LFC expression. (E) Scatterplot comparing log2 fold change of both analyses (vs GFP-negative and uninfected cells). Labeled genes represent enriched (blue) or depleted (red) genes in both analyses greater than 15% LFC.

**Fig S3. Genes identified that potentially facilitate HIV-1 infection**

Scatterplot of all genes depleted in analyses comparing GFP-positive cells against **(A)** GFP-negative cells and against **(B)** uninfected cells.

**Fig S4. Genes identified that potentially restrict HIV-1 infection**

Scatterplot of all genes enriched in analyses comparing GFP-positive cells against **(A)** GFP-negative cells and against **(B)** uninfected cells.

**Fig S5. Assessing knockdown of DDR factors in MDMs during HIV-1 infection**

**(A)** mRNA expression for each targeted gene indicated was measured by qPCR following a double-treatment of 100 nM siRNAs in MDMs. **(B)** siRNA knockdown of SAMHD1 was validated by western blot. MDMs from 3 different donors were infected with NL4-3 BaL/G after pre-treament with siRNAs and 3 dpi, supernatants were measured for p24^gag^ by ELISA. **(C)** Proviral integration measured by Alu-PCR. **(D)** Paired donor experiments were normalized to SAMHD1 knockdown and expressed as a percentage of integration. **(E)** 2-LTR circle formation by qPCR. Data are displayed as the means ± SEM with each point representing a different MDM donor.

**Fig S6. BLM knockdown does not affect SAMHD1 expression and SAMHD1 phosphorylation**

**(A)** Expression of SAMHD1 (green) assessed by Western blot in MDMs infected with NL4-3 BaL after pre-treatment with siRNAs (top x-axis). **(B)** Expression of phosphorylated SAMHD1 at Thr592 (green) assessed by Western blot. **(C)** Samples expanded to include uninfected primary CD14+ monocytes, THP-1 cells, and CHME3 cells for SAMHD1 expression and **(D)** phosphorylated SAMHD1 at Thr592.

Tables

Table S1. SmartPool siRNA Catalog Numbers

| **GENE** | **CATALOG** **#** |
| --- | --- |
| Control | D-001810-01-20 |
| BLM | L-007287-00-0005 |
| FANCM | L-021955-00-0005 |
| RAD51 | L-003530-00-0005 |
| RECQL5 | L-019338-00-0005 |
| RMI1 | L-014527-01-0005 |
| RMI2 | L-015684-01-0005 |
| SAMHD1 | L-013950-01-0010 |
| SLX4 | L-014895-00-0005 |
| TOP3A | L-005279-00-0005 |
| WRN | L-010378-00-0005 |
| Accell-Control | D-001950-01-05 |
| Accell-BLM | E-007287-00-0005 |

Table S2. qPCR Primer Sequences

| **GENE** | **FORWARD SEQUENCE** | **REVERSE SEQUENCE** |
| --- | --- | --- |
| GAPDH | CAAGATCATCAGCAATGCCT | AGGGATGATGTTCTGGAGAG |
| BLM | GCTCAGGAAAGTGACTCTCTG | TTCTTCTGGAGAAGGTGGAAC |
| FANCM | CAATCCCACATGGCCGAAATG | TCCGAGAGCTTTATGAGCTTC |
| RAD51 | TTTGGAGAATTCCGAACTGG | AGGAAGACAGGGAGAGTCG |
| RECQL5 | GCTCTTGGACAGGAGGCTGATA | GCATGGTAAGCCTTGGCGTTCA |
| RMI1 | GAGCAGTGGAGGTTTCAAGTA | CATGCCATGCAGCTAAAAGCC |
| RMI2 | GGCAGGGTAGTGATGGCGGAC | CCTGAACCACTCCCATCACCAT |
| SAMHD1 | TCACAGGCGCATTACTGCC | GGATTTGAACCAATCGCTGGA |
| SLX4 | GCTGAAGAAGGAACTGGATAGG | TCCTTCAGCTTCAGAACCATC |
| TOP3A | ATGAGGCGGAGAGAAGGACT | CTGCATCTGGAAATCATGAGCC |
| WRN | GCATGCACTTATCCCAAGCG | GTTGAAGTCCGCTGTCAGGA |
| Early | GCCTCAATAAAGCTTGCCTTGA | TGACTAAAAGGGTCTGAGGGATCT |
| Intermediate | CTAGAACGATTCGCAGTTAATCCT | CTATCCTTTGATGCACACAATAGAG |
| Late | TGTGTGCCCGTCTGTTGTGT | GAGTCCTGCGTCGAGAGATC |
| Alu/Gag | GCCTCCCAAAGTGCTGGGATTACAG | GTTCCTGCTATGTCACTTCC |
| 2-LTR | TGGTTAGACCAGATCTGAGCCT | AGGTAGCCTTGTGTGTGGTAGATCC |
| U5 LTR | CTAGAGATCCCTCAGACCCTTT | TTCAAGTCCCTGTTCGGGCG |
| PBS | CGCCCGAACAGGGACTTGA | GAGTCCTGCGTCGAGAGATC |
| psi | CGCAGGACTCGGCTTGCT | TTTGGCGTACTCACCAGTCG |
| cPPT | TAAAAGAAAAGGGGGGATTGGG | GTTTGTATGTCTGTTGCTATTATGTCTACT |
